# CD36 selection of 3D7 *Plasmodium falciparum *associated with severe childhood malaria results in reduced VAR4 expression

**DOI:** 10.1186/1475-2875-7-204

**Published:** 2008-10-09

**Authors:** Pamela A Magistrado, Trine Staalsoe, Thor G Theander, Lars Hviid, Anja TR Jensen

**Affiliations:** 1 Centre for Medical Parasitology at the Institute of International Health, Immunology and Microbiology, University of Copenhagen, Copenhagen, Denmark; 2 Centre for Medical Parasitology at the Institute of International Health, Immunology and Microbiology, Department of Clinical Microbiology, Copenhagen University Hospital (Rigshospitalet), Copenhagen, Denmark

## Abstract

**Background:**

A subset of the *Plasmodium falciparum *erythrocyte membrane protein 1 (PfEMP1_SM_) is involved in the cytoadherence of *P. falciparum*-infected red blood cells (iRBC) contributing to the pathogenesis of severe disease among young children in malaria endemic areas. The PfEMP1_SM _are encoded by group A *var *genes that are composed of a more constrained range of amino acid sequences than groups B and C *var *genes encoding PfEMP1_UM _associated with uncomplicated malaria. Also, unlike *var *genes from groups B and C, those from group A do not have sequences consistent with CD36 binding – a major cytoadhesion phenotype of *P. falciparum *isolates.

**Methods:**

A 3D7 PfEMP1_SM _sub-line (3D7_SM_) expressing VAR4 (PFD1235w/MAL8P1.207) was selected for binding to CD36. The protein expression of this parasite line was monitored by surface staining of iRBC using VAR4-specific antibodies. The serological phenotype of the 3D7_SM _parasites was determined by flow cytometry using malaria semi-immune and immune plasma and transcription of the 59 *var *genes in 3D7 were analysed by real-time quantitative reverse transcriptase-polymerase chain reaction (RT-PCR) using *var*-specific primers.

**Results:**

A selection-induced increased adhesion of 3D7_SM _iRBC to CD36 resulted in a reduced *var4 *transcription and VAR4 surface expression.

**Conclusion:**

VAR4 is not involved in CD36 adhesion. The current findings are consistent with the notion that CD36 adhesion is not associated with particular virulent parasite phenotypes, such as those believed to be exhibited by VAR4 expressing parasites.

## Background

Variant surface antigens (VSA) on the *Plasmodium falciparum*-infected red blood cells (iRBC) are involved in both cytoadherence contributing to disease pathology [1-8] and immune evasion which probably contributes to the severity and persistence of malaria infections [9-11]. Anti-VSA antibodies have been shown to contribute to protective immunity [12-16] and data from several studies indicate that some important targets for immunity seem to have restricted heterogeneity.

The *Plasmodium falciparum *Erythrocyte Membrane Protein-1 (PfEMP1) is the most studied VSA. PfEMP1 molecules are encoded by the *var *gene family comprising 50–60 highly diverse genes per haploid genome [17-22]. Any single parasite nuclei transcribes only one variant at a time, a phenomenon referred to as allelic exclusion or mutually exclusive expression [20,23-26]. Cultures of unselected 3D7 parasites predominantly express PfEMP1 mRNA species resembling those of parasites causing uncomplicated malaria (PfEMP1_UM_). The dominant serological phenotype (recognition by semi-immune and immune human plasma) changes to PfEMP1_SM _following selection of 3D7 using DynaBeads coated with IgG from semi-immune children [27]. This 3D7_SM _shows transcriptional upregulation and protein surface expression of one particular group A *var *gene, *var4 *(*PFD1235w/MAL8P1.207*)[28]. More recently, group A *var *genes, together with certain other B/A type were found to be transcribed in isolates from children with cerebral malaria, but not from isolates from equally highly parasitaemic patients without severe malaria syndromes [29].

Group A *var *genes are one of three major groups (A, B and C) of 3D7 *var *gene sequences categorized according to chromosomal location, gene orientation, domain structure of the encoded proteins and similarities in coding and non-coding regions. Group A contains seven genes encoding large PfEMP1s of about 400 kDa, with complex domain arrangements and three genes encoding PfEMP1s of about 150 kDa [30,31]. Unlike most other *var *genes from group B and C, the Group A *var *genes do not encode sequences thought to be consistent with binding to CD36, a major endothelial receptor for iRBC sequestration [31]. It should be noted however that the correlation between severity, binding phenotype and *var *gene expression is not clear-cut [4,32].

In this study, changes in the transcription of all *var *genes and surface expression levels of VAR4 as a result of drifting and changes following selection for binding of 3D7_SM _to CD36 was investigated.

## Methods

### Parasites

All parasites used in this study were derived from the *P. falciparum *isolate, 3D7 and were cultured in O Rh+ RBCs as described previously [33]. Three 3D7 sub-lines were used, namely: 3D7_SM-CD36_,3D7_SM-drift _and 3D7_UM_.

3D7_SM-CD36 _was obtained by bio-panning 3D7_SM _on Chinese hamster ovary (CHO) cells expressing CD36. 3D7_SM _has previously been selected from 3D7 using a pool of plasma from semi-immune Ghanaian children [27,28]. To select for a CD36-binding 3D7_SM _population, the method described in [27] was followed. Gelatine enriched late-stage 3D7_SM _was panned on a monolayer of wild-type CHO cells. Unbound iRBC and uninfected RBC (uRBC) were then panned on CHO cells transfected with human CD36. The monolayer was washed repeatedly to remove unbound iRBC and uRBC. To allow bound 3D7_SM _parasites to reinvade and grow, culture medium and uRBC were added to the monolayer of CD36-transfected CHO cells and incubated overnight.

3D7_SM _was allowed to drift without routine selection using the Ghanaian plasma pool to obtain the subline 3D7_SM-drift_. For consistency, 3D7_SM-drift _and unselected 3D7 were late-stage enriched by gelatine flotation [34] every time 3D7_SM-CD36 _was selected for CD36 binding. The gelatine-enriched unselected 3D7 was termed 3D7_UM _and was serologically similar to parasites causing uncomplicated malaria [27].

The genotypic identity of the 3D7 isolate and its derived sub-lines were confirmed by PCR at the polymorphic *msp1*, *msp2*, and *glurp *loci [35].

### Immuno-staining of live cells and flow cytometry

Immuno-staining and flow cytometry were carried out as described [28,33] with minor modifications. To monitor VAR4 expression on the surface of live cells, late stage-iRBC were enriched by magnet-activated cell sorting (MACS, Miltenyl BioTec, Bergisch Gladbach, Germany)[36,37]. MACS purified and ethidium bromide-labeled iRBC (2.5 × 10^5^) was incubated for 1 hr in 20 μL mouse sera or 40 μL rabbit sera raised against the CIDR1α or DBL5δ domains of VAR4[28] depleted for anti-human RBC antibodies prior to use. To detect surface bound antibodies, the iRBC were sequentially exposed to 100 μL of 1:25 goat anti-mouse or anti-rabbit Ig (DAKO), biotinylated anti-goat Ig (DAKO) and 1:1,000 fluorescein isothiocyanate (FITC)-conjugated streptavidin (BD Pharmingen) for 30 minutes each. For serological phenotyping, 20 human plasma samples from Magoda village in Tanzania with high malaria transmission were used to determine the recognition profile of the various parasite lines. Out of the 20 Magoda plasma samples, 10 came from children aged 3–5 years and 10 came from adolescents and adults aged 12–63 years. Staining of the iRBC by human IgG was done by incubating 2.5 × 10^5 ^MACS purified ethidium bromide-labeled iRBC in 5 μL human plasma for 30 minutes and then sequentially exposed to 100 μL of 1:50 biotinylated rabbit anti-human IgG (DAKO) and 1:2000 FITC-conjugated streptavidin (DAKO) for 30 minutes each. All immuno-stained samples were analysed in a Coulter EPICS XL-MCL flow cytometer (Coulter Electronics, Luton, UK) or in a FACScan (Becton Dickinson). Data were analysed in WinMDI software  and WinList version 5.0 (Verity Software House, Inc., Maine, USA). Student's t-test was used to determine whether the samples compared were significantly different (P = ≤0.05).

### RNA extraction, cDNA synthesis and Quantitative Real-Time PCR

RNA extraction, cDNA synthesis and quantitative real-time PCR were done as previously described [28,38]. Trophozoite/schizont-iRBC (36–48 h after invasion) were isolated from mixed stage *in vitro *cultures by exposure to a strong magnetic field as described in the previous section. To obtain ring stage parasites (30 h), the late stage enriched iRBC were cultured overnight. The ring stage has previously been shown to be optimal for studies of *var *gene transcription [38] and is therefore used for RNA extraction. Total RNA was purified using Trizol (Invitrogen) and treated with DNAseI (Invitrogen) for 15 min at 37°C. Superscript II was used to reverse transcribe DNA-free RNA primed with random hexamer primers (Invitrogen) at 25°C for 10 min, 42°C for 50 min followed by 70°C for 15 min. Quantitative real-time PCR was done using a Rotorgene thermal cycler system (Corbett Research, Motlake, Australia) and real-time PCR-optimized and gene-specific primers for each of the 59 full-length *var *genes in the *P. falciparum *3D7 genome [28,38]. Reactions were performed in 20 μl volumes using QuantiTect SYBR Green PCR master mix (Qiagen, Merck Eurolab, Albertslund, Denmark) and 1 μM primers. Quantification was done using Rotorgene software version 4.6. The housekeeping gene *seryl-tRNA synthetase*, which shows a uniform transcription profile in different parasite isolates and an unchanged pattern throughout the parasite life cycle, was used as an endogenous control as previously described [38] and used for calculations of percent measured transcript out of total *var *transcipts by the ΔΔCT method (User Bulletin no. 2, Applied Biosystems).

### CD36 binding assay

The CD36 binding assay was done as described in detail elsewhere [27]. To radiolabel parasites, cultures were incubated overnight in 10% non-immune Danish plasma in RPMI 1640 in the presence of ^3^H-hypoxanthine. Monolayers of wild type, CD36- and CD54-transfected CHO cells and transformed human bone marrow endothelial cells (TrHBMEC) were grown in 96-well microtiter plates (Nunc, Roskilde, Denmark). Late stage enriched iRBC (100 μl, 1 × 10^7 ^iRBC/ml) were added to the CHO cell monolayer and incubated for 1 hour at 37°C. After removal of unbound iRBC, the number of iRBCs adhering to the CHO cells was determined by liquid scintillation spectrometry.

## Results

### Reduced expression of VAR4 following selection for CD36 binding

**Figure 1:**
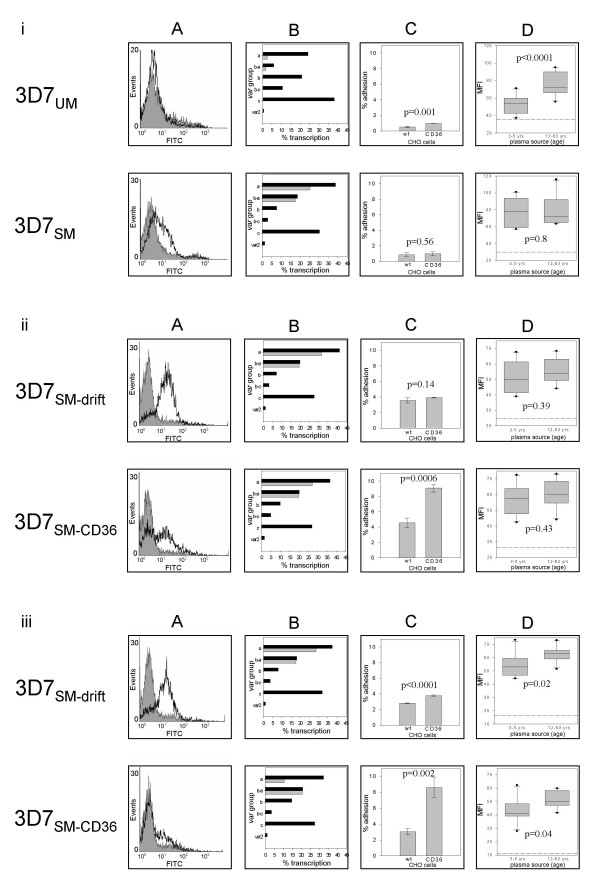
**Serological phenotypes, transcription and adhesion profiles of four 3D7 sub-lines, 3D7_SM_, 3D7_UM_, 3D7_SM-drift _and 3D7_SM-CD36_**. Panel (i): Parental 3D7_UM _and 3D7_SM_, 3D7_SM-_drift and 3D7_SM-CD36 _at the beginning of the experiment and following three (ii) and five (iii) rounds of gelatine flotation or selections for binding to CD36. Panel (A) shows the surface expression of VAR4 (open histograms) detected by flow cytometry using VAR4 anti-CIDR1α mouse (i) and rabbit anti-CIDR1α sera (ii and iii). Reactivity with pre-immunisation sera is shown for comparison (shaded histograms). Histograms are from one out of three replicate assays described in the Figure 2 legend. Panel (B) shows the total percentage of ring-stage transcripts of each *var *gene group out of total *var *transcripts (black bars). Grey bars show the percentage transcription of *var4 *and *PFF0010w *a group A and B/A gene highly upregulated in the 3D7_SM _compared to the 3D7_UM _sub-line. Panel C shows the percentage of iRBC adhering to wild type and CD36-transfected Chinese hamster ovary cells (CHO) compared with the total number of iRBC added to the plate. Bars are means of triplicate wells showing standard deviations and p values. Panel D shows t-tests comparing the recognition (average mean fluorescence intensities, MFI) of the different parasite sub-lines by plasma from 10 children (3–5 years) and 10 adults (12–63 years). The 95% confidence interval, standard deviations, outliers and p values are shown. p values ≤ 0.05 were considered significantly different. The cut-off (reference line) was defined as the mean MFI values obtained using plasma from three Danish donors never exposed to malaria plus two times standard deviation.

To determine the effect of CD36 selection on VAR4 surface expression, mouse or rabbit antibodies against the CIDR1α domain of VAR4 were used to stain three 3D7 sub-lines – 3D7_UM_, 3D7_SM-CD36 _and 3D7_SM-drift _and the parasite protein surface expression subsequently analysed by flow cytometry (Figure 1A). In agreement with what has been previously shown [28], 3D7_SM _surface expressed VAR4, whereas 3D7_UM _did not show any surface staining for VAR4 at the beginning of the experiment (Figure 1i-A). Similarly 3D7_SM _showed high *var4 *transcription, whereas 3D7_UM _only transcribed *var4 *at a very low level (Figure 1i-B).

**Figure 2:**
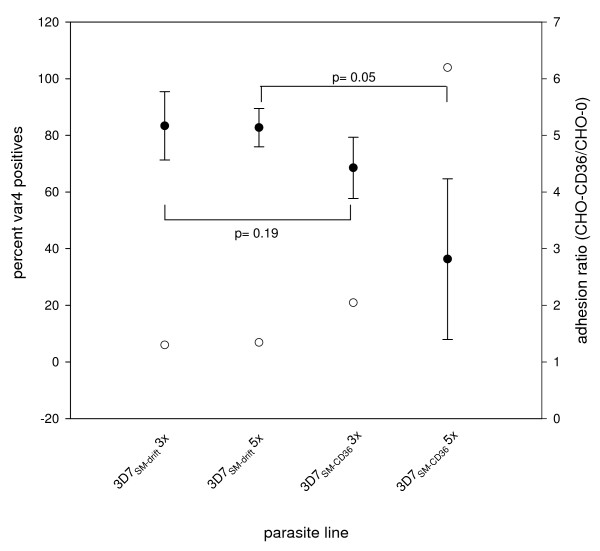
**VAR4 surface expression in 3D7_SM-_drift and 3D7_SM-CD36 _in 3 replicate experiments correlated with CD36 adhesion**. Left Y-axis, solid circles show mean percent VAR4 positives of 3D7_SM-_drift and 3D7_SM-CD36 _following three (3×) and five (5×) rounds of gelatine flotation or selections for binding to CD36 of three replicate experiments. Standard deviations are shown as error bars. The 3D7 sublines were not frozen nor thawed for the first replicate experiment prior to determination of VAR4 surface expression. For the second and third replicate experiments there was prior freezing, thawing, and *in vitro *culture of the 3D7 sublines. p values ≤ 0.05 were considered significantly different. Right Y-axis, open circles show adhesion ratio of the different 3D7 sublines. Adhesion ratio is calculated as percent adhesion to CHO-CD36 over percent adhesion to CHO-wild type.

As a control, the VAR4 expressing 3D7_SM _was left in culture for 44 generations from the day of the last antibody selection with gelatine flotation being carried out approximately every nine generations resulting in the 3D7_SM-drift _sub-line. Both 3D7_SM-CD36 _and 3D7_SM-drift _expressed VAR4 on the surface all throughout the experiment, but with the 3D7_SM-CD36 _sub-line showing a reduced level from the third panning (Figure 1ii-A and Figure 2). However, this reduction in VAR4 surface expression is not statistically significant (p = 0.19, Figure 2). Following the fifth selection, VAR4 surface expression was borderline significantly lower in 3D7_SM-CD36 _compared with that in 3D7_SM _(p = 0.05, Figure 2). Similarly, *var4 *gene transcription in the 3D7 sublines following the fifth selection was markedly lower in 3D7_SM-CD36 _(10%) as compared to the level of transcription in 3D7_SM _(28%) (Figure 1iii-B). By contrast the transcription level of the group B/A *var *gene *PFF0010w *(formerly known as *MAL6P1.316*)remained the same for both sub-lines (3D7_SM-drift _and 3D7_SM-CD36_) throughout the entire experiment (Figure 1ii-B and 1iii-B).

By using Western blotting for detection of VAR4 expression in 3D7_SM-CD36_and 3D7_SM-drift _sub-lines, a distinct 400 kDa VAR4 band was expressed following all selections, but it was not possible to accurately quantify the amount of the protein due to the insufficient sensitivity of the technique. However, the transcriptional data and flow cytometry of live immuno-stained parasites indicate that the 3D7_SM _parasites which bind to CD36 have reduced levels of VAR4 PfEMP1 surface expression (Figure 1i–iii and Figure 2).

### Increased adhesiveness to CD36

To test whether the CD36 bio-panning increased the adhesiveness of 3D7_SM _to the CD36 molecule, 3D7_UM_, 3D7_SM-CD36 _and 3D7_SM-drift _binding assays were done using CD36 and CD54 (also known as intercellular adhesion molecule or ICAM-1) expressing CHO cell lines and TrHBMEC. Due to high background adhesion of iRBC to wild type CHO cells only iRBC showing more than 8% binding to CHO-CD36 as seen following the third and fifth panning (Fig 1ii–iiiC) of 3D7_SM-CD36 _were considered CD36 binders. Increased binding of 3D7_SM-CD36 _to CD36 correlated well with reduced VAR4 expression and gene transcription (Figure 1A to 1C and Figure 2). The adhesion of 3D7_SM-drift _to the CHO-CD36 was low in all selections (Figure 1iC–iiiC). Binding of 3D7_SM-CD36 _to CHO-CD54 or TrHMBEC following bio-panning on CD36 did not differ from that of 3D7_SM-drift_, indicating that bio-panning on CD36 was specifically selecting for parasites binding to the CD36 receptor.

### Serological phenotypes of 3D7_SM _selected for CD36 binding

Consistent with previous published data [27,28], unselected 3D7 predominantly expressed PfEMP1_UM _and were better recognized by adults (12–63 years) than by children (3–5 years) (p < 0.0001) while 3D7_SM _selected using IgG from semi-immune children was equally well recognized by plasma from both groups of individuals (p = 0.8) (Figure 1i-D). Three to five rounds of gelatine flotation of 3D7_UM _did not change the recognition pattern of this subline (data not shown). Following the third round of selection 3D7_SM-drift _and 3D7_SM-CD36 _had similar phenotypes to that of the original 3D7_SM _(Figure 1i–iiD). Both sub-lines became significantly better recognized by adult plasma than by children's plasma after the fifth round (44 generations) of gelatine flotation (p = 0.02) or CD36 selection (p = 0.04, Figure 1iiiD) having serological profiles correlating slightly better with the baseline 3D7_UM _compared to the 3D7_SM_. Although borderline, this would indicate that both 3D7_SM-drift _and 3D7_SM-CD36 _have gradually shifted from expressing PfEMP1_SM_-type antigens to expressing PfEMP1_UM_-type antigens.

### Stability of VAR4 surface expression in 3D7_SM _left to drift

**Figure 3:**
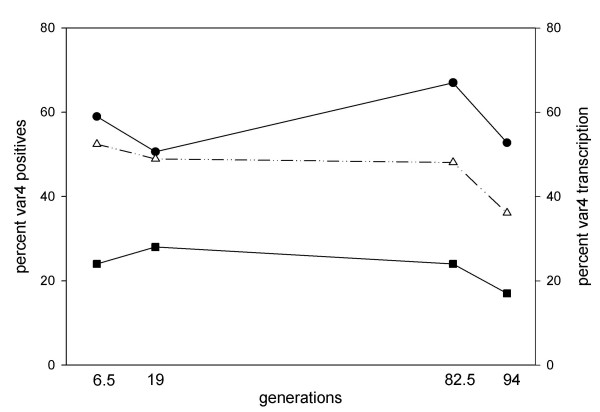
**Changes in VAR4 surface expression and transcription of the 3D7_SM _subline left to drift (3D7_SM-drift_)**. Left Y-axis, percentage of infected blood cells positive for VAR4 using flow cytometry and VAR4-specific CIDR1α (closed circle) and DBL5δ (open triangle) rabbit antibodies. Right Y-axis, percentage *var4 *gene transcription in ring stage (closed square) parasites calculated as: (*var4 *absolute copy number/∑copy number of all 3D7 *var *genes) × 100%. Fluorescence of 3D7_SM-drift _stained with pre-immune serum was used to separate positive cells from cells with background fluorescence.

In the absence of CD36 selection VAR4 is stably expressed on the surface of 3D7_SM-drift _for 44 generations – equivalent to five rounds of gelatine flotation (Figure 1iii-A). To analyse this further 3D7_UM _and 3D7_SM _were cultured without gelatine flotation or CD36 selection and monitored VAR4 surface expression and *var4 *transcription at specified time points for 94 generations. To avoid assay-to-assay variation, RBC infected with the two parasite sub-lines were frozen down at each sampling point. Frozen iRBC were thawed and cultured for another six generations and then assayed on the same day. Almost no VAR4 surface expression and *var4 *gene transcription was seen in the parental 3D7_UM_, whereas 3D7_SM_-iRBC showed high and stable surface expression of VAR4 as well as gene transcription for at least 94 generations (Figure 3). Using the percentageVAR4 positive iRBC and the ring-stage *var4 *transcription level at generation six (first sampling point) and 94 (last sampling point) the switch off-rate was calculated to be less than 0.005% (Figure 3). Alternatively, with a switch off-rate that is virtually 0, the minor differences observed at the transcription level may simply be due to experimental errors. Parallel to this, the two 3D7 sub-lines without prior freezing and thawing were assayed at every ~9th generation and similarly found VAR4 expression and transcription to be stable for over six months in continuous *in vitro *culture.

## Discussion

A previous study has shown that *var4*, a group A *var *gene, is highly transcribed and surface expressed in 3D7 parasites expressing PfEMP1_SM _(3D7_SM_) compared to 3D7 expressing PfEMP1_UM _(3D7_UM_), suggesting an association between VAR4 and severe malaria [28]. CD36 binding has in some studies been associated with disease severity. In a study in Kenya, a mutation in the CD36 gene among children has been associated with protection from severe malaria [39]. In addition, *P. falciparum *isolates from Thai adults suffering from severe but not cerebral malaria had higher CD36 adhesion than those from uncomplicated malaria patients [40]. In contrast, several studies are consistent with the notion that CD36 binding is not associated with particular virulent parasite phenotypes [27,28,30-32,41-43]. Robinson *et al *[31] has demonstrated in a heterologous expression assay that the CIDR1 region of VAR4 did not bind to CD36. There might be a conformational difference between the heterologous recombinant protein and the native protein presented on the iRBC or binding could be through a different domain. However, it remains that the current data on VAR4 expression on iRBC and CD36 binding phenotype support the prediction made by Robinson *et al *[31]. The current study has shown that VAR4 protein expression and *var4 *mRNA transcription are reduced following selection for CD36 binding, lending further support to the hypothesis that PfEMP1_SM _antigens such as VAR4 are not involved in CD36 binding.

However, it is important to note that the correlation between severe malaria, the inability to bind to CD36 and group A *var *gene expression is not clear-cut [4,31]. ICAM-1 binding may be associated with cerebral malaria as shown by previous studies [43-45]. But as demonstrated by Smith *et al *[4], at least two group B PfEMP1 that bind to ICAM-1 also bind to CD36. In addition, not all severe malaria isolates express group A *var *genes [42].

Selection of 3D7_SM _for binding to CD36 lead to a reduced *var4 *gene transcription, but did not show any significant changes in other *var *genes as such. *PFF0010w*, a group B/A *var *gene upregulated in 3D7_SM _together with *var4 *was previously predicted to be a non-CD36 binder based on its CIDR1 sequence [31]. In the current study however, selection on CD36 did not reduce *PFF0010w *transcription as would be expected. The reason for this is that PFF0010w is probably not expressed on the surface of 3D7_SM _parasites as indicated by one distinct population of VAR4 expressing parasites in 3D7_SM _and 3D7_SM-drift _(Figure 1i–iiiA). Previous studies have shown that only one PfEMP1 is expressed on the surface at a time [24,46] so these VAR4 expressing parasites are less likely to be expressing PFF0010w. However, a PFF0010w-specific antibody is needed to confirm this.

Although Group B and C *var *genes have been linked to CD36 binding [30,31], new protein bands were not detected in 3D7_SM-CD36 _using Western blots and anti-ATS antibodies compared to the protein band pattern seen in extracts of 3D7_SM_. Furthermore, the serological phenotype of 3D7_SM-drift _and 3D7_SM-CD36_remained similar when analysed by flow cytometry. These results might be explained by the fact that only 8% of the 3D7_SM-CD36 _parasites bound to the CD36 receptor while transcription and serological analysis was done on the entire parasite population. With the present set up it is difficult to determine the exact *var *genes involved in the CD36 adhesion of 3D7_SM-CD36 _although a slight increase in the total transcription level of group B *var *genes was noted in this sub-line at the end of the experiment (Figure 1iii-B). In order to determine the PfEMP1 involved in CD36 binding, both transcription and protein expression data are required.

*Var *gene transcription does not always reflect phenotypic expression as observed in this study, where VAR4 protein expression was poorly correlated with *var4 *transcription after the third CD36 panning (Figure 1ii-A and 1B). However, the fifth CD36 panning resulted to a higher correlation between *var4 *transcription and protein expression (Figure 1iii-A and 1B) most likely due to the more homogenous population as indicated by the dominant peak of VAR4 negative parasites in the histogram (Figure 1iiiA).

Studies have shown that *var *gene transcription peaks during the ring stage at 3–18-hour post invasion [47,48] and that full length *var *transcript is not seen at all by Northern blotting at 24–34-hour post invasion [49]. In the current study, a 30-hour post invasion ring stage was used to measure *var *gene transcription. At 30 hours, the parasites appeared as non-pigmented thick ring forms under the microscope. An improved quantification of transcript abundances of each *var *gene obtained by quantitative real time PCR and the use of cross intron primers show that although the dominant full length transcript peaks at the ring stage, it remains dominant through out the life cycle [50].

In the absence of immune pressure *var *genes have been reported to have variable transition rates *in vitro*. There have been reports of transition rates as low as 0.025% [51] to as high as 2.4% [52] per generation. In the absence of CD36 selection and immune pressure, VAR4 surface expression is stable for more than six months in continuous *in vitro *culture with a transcription off rate of less than 0.005%. For a PfEMP1 implicated in severe disease and expected to dominate in a host with limited immunity, it may not be surprising that VAR4 appears to have a low switching rate. Frank *et al *[53] have recently observed that, in the 3D7 isogenic line NF54, sub-telomeric *var *genes have higher off rates of approximately 1% compared with central *var *genes with 0 to 0.3% off rates. However, Frank *et al *[53] did not observe any activation of *upsA *genes in their experiments hence the high switch off rates of the sub-telomeric *var *genes reported were that of the *upsB *promoter type. *var4 *is a sub-telomeric *var *gene with an *upsA *promoter suggesting the possibility that at least for some Group A *var *genes the switch rate might be comparable to that of central *vars*.

Both 3D7_SM-drift _and 3D7_SM-CD36 _drifted away from a serological SM phenotype at prolonged *in vitro *culture to a more UM like phenotype. However, surprisingly the 3D7_SM-drift _remained positive for VAR4 surface expression raising the question whether VAR4 is the only PfEMP1 molecule contributing to the SM phenotype of the original antibody selected parasite, 3D7_SM_. To clarify this, in the current study, 3D7_UM _was selected using VAR4 specific antibodies and found that only some and not all semi-immune plasma from children recognized 3D7_VAR4_. This is an important finding because it points to the possibility that VSAs other than VAR4 might contribute to the serological phenotype of 3D7_SM_.

## Conclusion

This study shows that increased CD36 adhesiveness of 3D7_SM-CD36_resulted in a reduction in VAR4 surface expression. In the absence of CD36 selection and immune pressure, VAR4 expression by 3D7_SM _parasites is stable for at least six months while the population's serological phenotype drifts towards the UM phenotype. Results of this study shed more light on the characteristics of 3D7 parasites expressing VAR4 – a PfEMP1 previously associated with severe malaria phenotypes.

## Competing interests

The authors declare that they have no competing interests.

## Authors' contributions

PAM participated in the study design, carried out parasite work, flow cytometry experiments, data analysis and writing of the manuscript. TS participated in the study design, performed CD36 selection and binding assays, as well as serological phenotyping and data analysis. TGT helped in the study design and in finalizing the manuscript. LH helped in finalizing the manuscript. ATRJ participated in the study design, performed the RNA extractions, cDNA synthesis, all the Q-RT PCR experiments, analysed the data and helped drafting and writing the manuscript. All authors read and approved the final manuscript.
